# A 20-year bibliometric analysis of Fuchs endothelial corneal dystrophy: from 2001 to 2020

**DOI:** 10.1186/s12886-022-02468-x

**Published:** 2022-06-08

**Authors:** Feng Lin, Luoli Zhang, Yimin Wang, Dan Fu, Yuliang Wang, Xingtao Zhou

**Affiliations:** 1 Eye Institute and Department of Ophthalmology, Eye & ENT Hospital, Fudan University, Shanghai, 200031 China; 2 NHC Key Laboratory of Myopia (Fudan University); Key Laboratory of Myopia, Chinese Academy of Medical Sciences, Shanghai, 200031 China; 3 Shanghai Research Center of Ophthalmology and Optometry, Shanghai, 200031 China; 4 Shanghai Engineering Research Center of Laser and Autostereoscopic 3D for Vision Care, Shanghai, 200031 China; 5 Department of Ophthalmology, Shanghai General Hospital, Shanghai Jiaotong University School of Medicine, Shanghai, 20080 China

**Keywords:** Fuchs endothelial corneal dystrophy (FECD), Bibliometric analysis, Research hotspots, VOSviewer

## Abstract

**Purpose:**

The aim of this study was to identify trends and focuses in the field of Fuchs endothelial corneal dystrophy (FECD) research.

**Methods:**

A bibliometric analysis based on the Web of Science Core Collection was conducted. All publications related to FECD from 2001 to 2020 were extracted and analyzed. VOSviewer v.1.6.17 was used to construct a visualization map and evaluate the trends and focuses in FECD research.

**Results:**

A total of 1,041 publications were extracted. The rate of global publications has steadily increased. The United States produced the highest number of publications (461), the highest number of citations (18,757), and the highest H index (69). Melles GRJ published the highest number of papers (60), and Price FW had the highest number of citations (4,154) in the FECD research field. The highest number of publications came from the journal *Cornea* (279). Keywords were classified into four clusters: (1) corneal transplantation surgery, (2) surgical techniques and instruments, (3) corneal parameter measurement, and (4) genetic and molecular pathomechanisms. The average appearing years (AAYs) of the keywords were evaluated. Recently appearing keywords included “Tcf4 gene” (AAY of 2018.3), “ctg18.1” (AAY of 2017.2), “trinucleotide repeat expansion” (AAY of 2018.3), “rock inhibitor” (AAY of 2017.4), and “descemetorhexis” (AAY of 2017.4).

**Conclusions:**

The United States has a dominant position in FECD research. Although corneal transplantation surgery has been the most mainstream area of FECD research field for a long time, gene mutations such as the TCF4 CTG trinucleotide repeat expansion, nonsurgical interventions such as rho-associated kinase inhibitors, and newer surgical methods such as descemetorhexis without endothelial keratoplasty are potential research hotspots.

**Supplementary Information:**

The online version contains supplementary material available at 10.1186/s12886-022-02468-x.

## Introduction

Fuchs endothelial corneal dystrophy (FECD), a slow progressive disease, was first reported by Ernst Fuchs in 1910 [1]. It is the most common form of posterior corneal dystrophy which affects mainly the corneal endothelium and results in the loss of endothelial cells [2]. In the early stages of FECD, cornea guttae, a kind of excrescence, may appear on the Descemet membrane. In the late stages of FECD, complete loss of corneal endothelium function can lead to worsening stroma edema and epithelial bullae [3]. Finally, subepithelial scarring and corneal vascularization occurred.

Previous epidemiological surveys suggested a prevalence of cornea guttae in the United States of 4 to 7% [4, 5]. Considering the relatively high prevalence of cornea gutta which occasionally progresses to FECD, early prevention and treatment are particularly important. There are few nonsurgical treatments for FECD [6]. Current treatment relies mainly on corneal transplantation surgeries, such as penetrating keratoplasty (PK) and endothelial keratoplasty (EK) [7]. Despite the existence of many surgical interventions and relatively mature surgical techniques, various surgical complications and problems such as a shortage of corneal donors still exist. It is therefore urgent to explore new non-surgical interventions.

In recent years, the pathomechanisms of FECD have received more attention than before. Though its pathomechanisms have not been fully elucidated, some gene mutations related to FECD have been identified, such as the collagen type 8 α2 chain (COL8A2), transcription factor 4 (TCF4) and solute carrier family 4 member 11 (SLC4A11) [2]. It is worth noting that Koizumi N et al. introduced rho-associated kinase (ROCK) inhibitor eye drop for the management of FECD in 2013 [8]. This eye drop may yield insights into nonsurgical treatments for FECD.

The quantitative analysis method of bibliometrics uses mathematical and statistical methods to evaluate the impacts of publications, authors, journals, institutions, and countries. By mapping a knowledge domain, a bibliometric study can reveal the core structure of knowledge and predict research trends in an academic field [9]. Such analyses can provide data to inform policy-making and clinical guidelines.

This study aimed to conduct a bibliometric analysis of FECD over a 20-year period. The objective was to reveal the knowledge structure and identify research trends and potential focuses in the field of FECD research.

## Methods

### Data source and research process

**Fig. 1:**
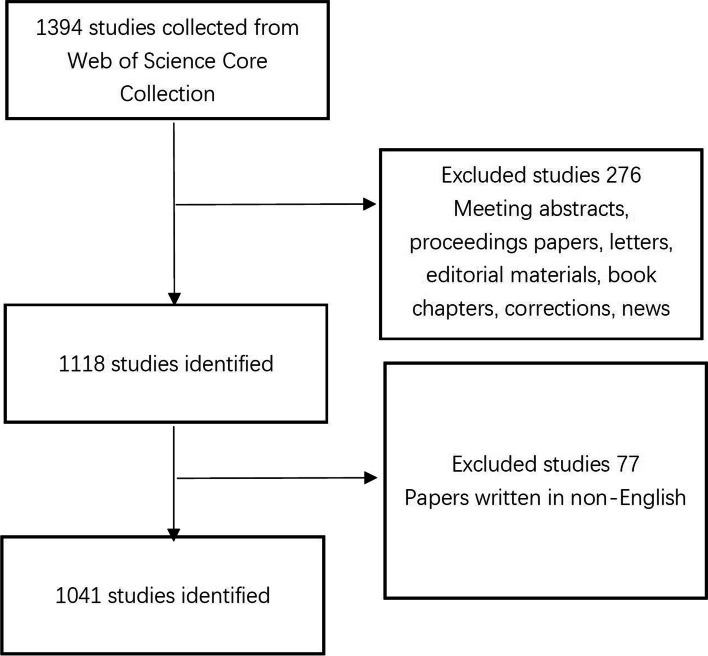
Workflow chart

The data source for this bibliometric study was the Science Citation Index-Expanded (SCI-E) in the Web of Science Core Collection (WoSCC). We conducted all searches on September 22, 2021. The search conditions were as follows: the retrieval topic was Fuchs dystrophy, the document types were articles or review articles, the timespan was 2001–01–01 to 2020–12–31, and the language was English. Ultimately, 1,043 publications were included in the bibliometric analysis. All details of the included publications (title, abstract, keyword, author, journal, country, citations, and H index) were collected from WoSCC. Top countries, top institutions, top authors and top journals with the highest number of publications in the field of FECD research were evaluated. In addition, all authors appearing in a paper were counted as an author during the process of evaluation of top authors. Co-occurrence analysis of keywords was further conducted by VOSviewer. Figure 1 shows the operating procedures.

### Analytic methods for bibliometric analysis

Relative research interest (RRI) represents the degree of worldwide attention to a certain research area. The RRI was obtained using the number of publications in a certain field divided by all publications in all fields within a year. The fitting curves for the growth trends were made using Microsoft Excel 2010 based on the prediction model f(x) = ax3 + bx2 + cx + d, which can predict future publication trends. The H index was obtained from the WoSCC; this index indicates that *h* papers have each been cited at least *h* times. The H index is useful for characterizing the scientific output of a given researcher or country [10]. The country collaboration map, which represents cooperation between countries, was created in the R programming language.

VOSviewer 1.6.17, a software tool used to construct and visualize bibliometric networks, was used in this study [11]. Maps based on co-authorship analysis of countries and authors were drawn. The size of a node in these maps is proportional to the number of collaborations. A network visualization map and an overlay visualization map were also drawn according to the co-occurrence of keywords. The size of a node in these maps is proportional to the frequency of keyword occurrence. In the network visualization map, all keyword nodes are classified into different clusters to reveal the knowledge structure of the field. In the overlay visualization, all keyword nodes are color coded according to the average appearing year (AAY). A lighter color represents a later AAY. This process can reveal potential research hotspots.

## Results

### Publication trend in the FECD research field

**Fig. 2:**
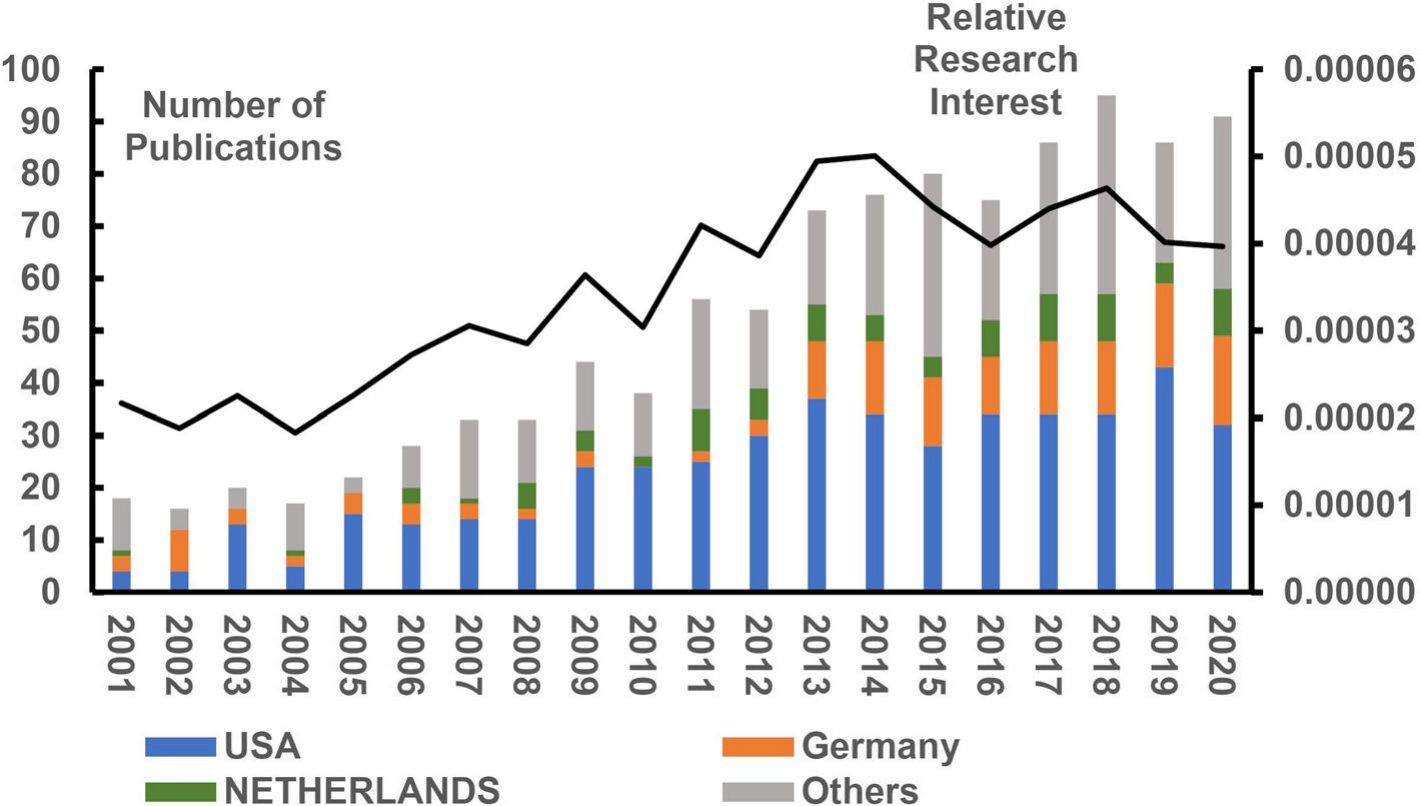
Publication trends for the past 20 years. The bar chart shows the number of publications, both worldwide and in the top three countries with the highest number of publications, for the past 20 years. The line chart shows the time course of relative research interest per year

The number of global annual publications in the field of FECD research has shown an upward trend in the past 20 years (Fig. 2). To further assess the degree of attention paid to FECD worldwide, Fig. 2 also shows the field’s annual RRI value. The RRI value rose from only 0.002% (the average value from 2001 to 2005) to 0.004% (the average value for the past 10 years). This indicates that overall global interest in FECD doubled, with the annual RRI value reaching its peak of 0.005% in 2014.

**Fig. 3:**
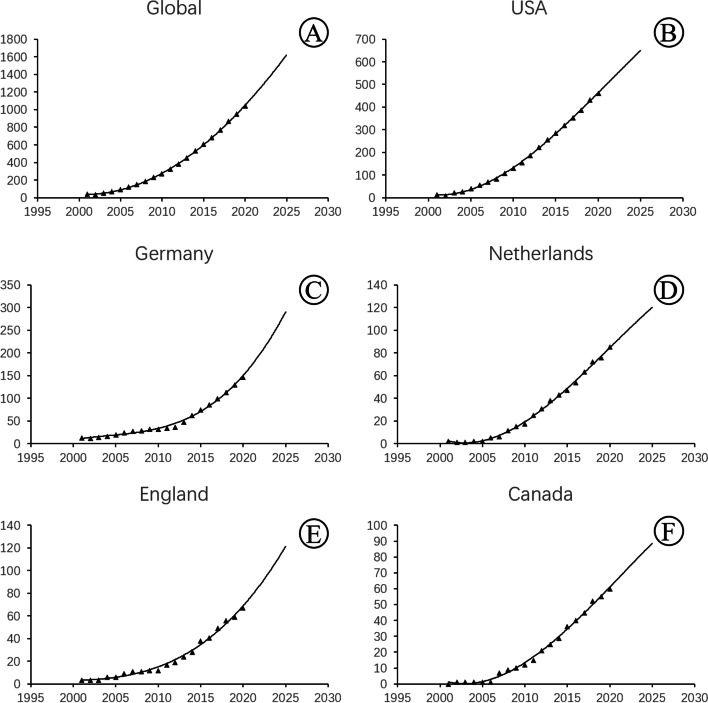
Model fitting curve of publication growth trends worldwide and in the top five countries with the highest number of publications in the field of FECD research. **A**. Global **B**. United States **C**. Germany **D**. Netherlands **E**. England **F**. Canada

Figure 3 shows the model-fitting curve for publications, which can be used to predict the number of publications in the next five years. The model was based on the number of publications in the past 20 years. It revealed that the number of publications, both worldwide and in the five countries producing the highest number of publications, has steadily increased over time and has shown a growth curve. The shape of the growth curves of England and Germany, in particular, indicated exponential growth. Overall, the rate of growth in the number of publications about FECD has increased rapidly in recent years.

### Top countries in the field of FECD research

Regarding the cumulative number of publications per country for the past 20 years, the United States ranked first (461 publications, 44.28%), Germany ranked second (147 publications, 14.12%), and Netherlands ranked third (85 publications, 8.17%) (Supplementary Fig. 1). Except in 2002, when it was surpassed by Germany, the United States also ranked first in the number of publications annually (Fig. 2). Regarding the total number of citations (Supplementary Fig. 1), the United States ranked first (18,757 total citations, 15,640 without self-citations), the Netherlands ranked second (3,773 total citations, 3,429 without self-citations), and Germany ranked third (3,687 total citations, 3,412 without self-citations). Regarding the H index (Supplementary Fig. 1), the United States ranked first (H = 69). There was little difference between Germany (H = 32) and the Netherlands (H = 31) in this regard, which ranked second and third, respectively. Although China ranked 11th regarding the cumulative number of publications (30 publications, 2.88%), the number of Chinese publications has risen at a surprising rate. Totally 26 of 30 papers were published between 2011 and 2020.

Supplementary Fig. 2 shows a map of co-authorship between the major countries in this research field. Five clusters were identified. Cluster 1 (red) includes Canada, England, India, Israel, Italy, Singapore, Australia, and the People’s Republic of China. Cluster 2 (green) includes the United States, France, Greece, Japan, South Korea, and Switzerland. Cluster 3 (blue) includes the Netherlands, Austria, Brazil, Poland, and Spain. Cluster 4 (yellow) includes Germany and Hungary. Cluster 5 (purple) includes Denmark and New Zealand. To visualize collaborations, a country collaboration map was made using the R language and is presented in Supplementary Fig. 3. It shows that the United States had the greatest number of collaborations with other countries.

### Top institutions in the field of FECD research

Johns Hopkins University ranked first among the top 20 institutions in FECD research, with the highest number of publications (75) accounting for 7.2% of total publications. The University of California system ranked second, with 6.1% of total publications (63 publications). The publications of the Netherlands Inst Innovat Ocular Surg ranked closely behind, with 5.9% of total publications (61 publications). Of the top 20 institutions, 14 were located in the United States, 3 were located in the Netherlands, 2 were located in Singapore, and 1 was located in Germany (Supplementary Fig. 4).

### Top authors in the field of FECD research

Melles GRJ published the highest number of papers (60) and ranked third in number of citations (3,115). Price FW had the second highest number of publications (51) but the highest number of citations (4,154). Price MO ranked third in the number of publications (49) and second in citations (4,035). Among the top ten authors, seven were from the United States, two were from the Netherlands, and one was from Germany (Table 1).

**Table 1:** Top ten Authors with the highest number of publications in the field of FECD research

Author	Country	Affiliation	No. of Publications	No. of Citations
Melles GRJ	USA	University of Alabama Birmingham	60	3115
Price FW	USA	Price Vis Grp	51	4154
Price MO	USA	Cornea Research Foundation of America	49	4035
Terry MA	USA	Natl Registry Emergency Med Tech	41	2539
Patel SV	USA	Mayo Clin Minnesota	40	1261
Dapena I	NETHERLANDS	Netherlands Inst Innovat Ocular Surg NIIOS	35	1669
Ham L	NETHERLANDS	Netherlands Inst Innovat Ocular Surg NIIOS	35	1933
Baratz KH	USA	Mayo Clin Minnesota	34	1335
Lass JH	USA	Case Western Reserve University	34	1450
Seitz B	GERMANY	University of Erlangen Nuremberg	31	1040

*FECD* Fuchs endothelial corneal dystrophy

The results of the co-authorship analysis are shown in Supplementary Fig. 5. All authors were divided into six clusters. The core of the cyan-blue cluster was Gottsch JD. The core of the purple cluster was Patel SV. The cores of the red cluster were Koizumi N and Okumura N. The core of the green cluster was Lass JH. The core of the yellow cluster was Dirisamer M. The core of the blue cluster was Melles GRJ.

### Top journals in the field of FECD research

The journal *Cornea* had the highest number of publications in FECD research (279) (Supplementary Fig. 6), accounting for 26.8% of total publications. The journals *Investigative Ophthalmology Visual Science* and *Ophthalmology* ranked second and third among the top 20 journals, accounting for 8.4% (87 publications) and 8.0% (83 publications) of total publications, respectively.

### Co-occurrence of keywords in the field of FECD research

**Fig. 4:**
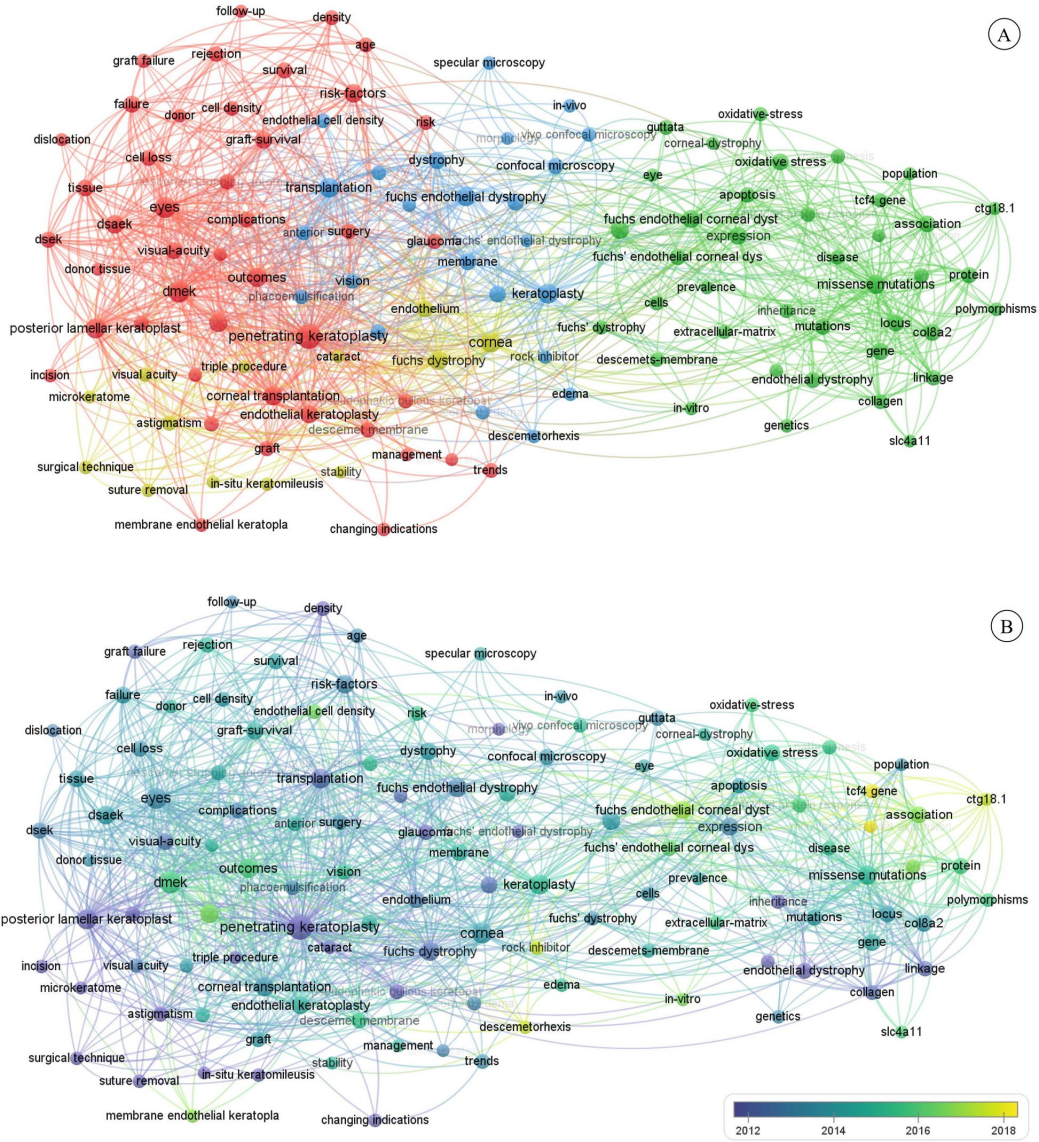
Co-occurrence analysis of keywords. The size of a node is proportional to the frequency of keyword occurrence. Two keywords connected by a line indicates that they appear in the same paper. A thicker line represents a closer relationship. **A** Mapping of keywords in the field of FECD research. Each color represents a single cluster. FECD: Fuchs endothelial corneal dystrophy. **B** Distribution of keywords according to AAY. Color intensity is related to the AAY, with a lighter color representing a later AAY. AAY: average appearing year

Using VOSviewer, 2,730 keywords in the field of FECD research were identified. When the minimum co-occurrence was set as 15, 120 keywords that met the threshold were extracted. Using network visualization, all keywords were classified into four clusters (Fig. 4A). The red cluster represented corneal transplantation surgery and included keywords such as “DMEK,” “DSEK,” and “penetrating keratoplasty.” The yellow cluster represented surgical techniques and instruments and included keywords such as “surgical technique,” “suture removal,” and “microkeratome.” The blue cluster represented corneal parameter measurement and included keywords such as “confocal microscopy,” “morphology,” and “optical coherence tomography.” The green cluster represented genetic and molecular pathomechanisms and included keywords such as “missense mutations,” “expression,” and “oxidative stress.”

With the overlay visualization, all keywords were further color coded according to their AAY (Fig. 4B). Potential hotspots in FECD research were identified by the intensity of color. Yellow-colored keywords, such as “tcf4 gene” (AAY of 2018.3), “ctg18.1” (AAY of 2017.2), “trinucleotide repeat expansion” (AAY of 2018.3), “rock inhibitor” (AAY of 2017.4), and “descemetorhexis” (AAY of 2017.4) were the most recent areas of interest in the field of FECD research.

## Discussion

### Research trends

As the RRI showed, the amount of research focusing on FECD has increased in recent years. Global publications have grown steadily for the past 20 years and, according to the prediction model, will continue to increase in the next five years. In addition, the rate of growth increased over time, with England and Germany in particular showing exponential growth. Their growth curves flat at the start of the study period but have curved sharply in recent years.

Based on the number of publications, number of citations, and H index of each country, the United States, Germany, and the Netherlands were the three most influential countries in FECD research. In addition, the United States engaged in the highest number of collaborations with other countries. This could be attributed to two factors. First, FECD is much more prevalent in Caucasians [3]. Approximately 4 to 7% of subjects in the United States had corneal guttae [4, 5]. In contrast, the prevalence of cornea guttae in the Japanese subjects was found to be only 3.7 to 4.1% [12, 13]. Second, the United States is equipped with advanced surgical techniques for treating FECD. Corneal transplantation surgery is currently the main treatment for FECD; approximately 185,000 of these procedures were performed worldwide each year, 34% of which were in the United States [14]. FECD was the reason for up to 39% of all corneal transplantation surgeries [14]. The number of transplantation surgeries for FECD in the United States is far greater than in other countries, which enables many clinical studies of FECD to be carried out there. This contributed to the United States’ great influence in the field of FECD research.

Based on an analysis of the institutions that produced the highest number of publications, this study revealed that Johns Hopkins University was the most productive research institution. Notably, 15 of the top 20 institutions were in the United States. This indicates the dominance of the United States in the field of FECD research.

Through an analysis of the authors with the highest number of publications, this study revealed that Melles GRJ, Price FW, and Price Mo were the most productive authors in FECD research. Notably, Price FW’s and Price Mo’s citations numbered over 4,000, far ahead of those of other authors. In addition, these three authors were committed primarily to clinical research on FECD, and they all made significant contributions to the development of EK. Melles GRJ contributed to the transition from PK to EK [15]. More importantly, he invented descemet’s membrane endothelial keratoplasty (DMEK) [16]. Price FW and Price Mo pioneered descemet’s stripping endothelial keratoplasty (DSEK). Price FW introduced techniques to promote donor tissue adherence [17], and Price Mo introduced the use of pre-dissected corneal grafts from eye banks [18].

*Cornea*, *Investigative Ophthalmology Visual Science*, and *Ophthalmology* were the three most influential journals in FECD research. *Cornea* had the highest number of publications on the subject, with more than the second and third highest numbers of publications combined. This indicates *Cornea*’s high output in the field of FECD research. In short, papers in the field of FECD research can be retrieved mainly from the three above-mentioned journals.

### Research focuses

Based on the network visualization map, we identified four research clusters: corneal transplantation surgery, surgical techniques and instruments, corneal parameter measurement, and genetic and molecular pathomechanisms. Corneal transplantation surgery is currently the main treatment for FECD. The most frequently occurring keyword, “penetrating keratoplasty”, appeared in publications 239 times. However, we identified potential research hotspots focused on the genetic pathomechanisms of FECD, nonsurgical interventions, and newer surgical methods with the overlay visualization map.

TCF4 gene mutation, research on which was indicated by keywords including “Tcf4 gene” (AAY of 2018.3), “ctg18.1” (AAY of 2017.2) and “trinucleotide repeat expansion” (AAY of 2018.3), refers to CTG trinucleotide repeat expansion [19]. TCF4 gene, which can encode E-2 protein, is a kind of transcription factor belonging to the basic helix-loop-helix family [20]. TCF4 gene mutation in FECD is CTG trinucleotide repeat expansion in the third intron of TCF4 gene on chromosome 18q. The length of the CTG repeat is correlated with the clinical severity of FECD [19]. Multiple gene mutations, such as COL8A2, SLC4A11, ATP/GTP binding protein like 1 (AGBL1) and lipoxygenase homology domain 1 (LOXHD1) gene mutations, are associated with FECD [2]. However, these gene mutations together could only account for a minority of FECD subjects. As the most common gene mutation in FECD, TCF4 CTG trinucleotide repeat expansion accounted for up to 79% of FECD subjects in the Caucasian population [21]. Nowadays, several pathogenic mechanisms regarding TCF4 CTG trinucleotide repeat expansion were proposed. First, it may lead to RNA-mediated toxicity. As the CTG repeats are transcribed to RNA, these RNA accumulate in the nucleus and sequesters RNA binding protein such as splicing factor muscleblind-like (MBNL) proteins, contributing to missplicing of pre-mRNA. [22] Second, the CTG repeats may directly alter the expression of the Tcf4 gene [23, 24]. Third, the peptides derived from the CTG repeats may induce cell toxicity. The CTG repeats will be transcribed into CUG and CAG RNA transcripts and further translated into toxic peptides without an AUG start codon [25]. Understanding the pathogenic mechanisms of CTG expansion-mediated FECD may help developing gene therapies targeting FECD. Nowadays, the clustered regularly interspaced short palindromic repeats/CRISPR-associated system 9 (CRISPR/Cas9) mediated gene editing techniques have been used in multiple repeat expansion-mediated diseases [26]. The techniques for FECD could reduce repeats containing RNA transcripts in the myotonic dystrophy type 1 (DM1) cells [27]. However, genes editing techniques for FECD are yet to be published. In addition, there is still a lack of CTG expansion-mediated FECD animal models [20]. All researches focusing on the pathogenic mechanisms regarding TCF4 CTG trinucleotide repeat expansion rely on tissue or cell culture.

ROCK inhibitor, research on which was indicated by the keyword “rock inhibitor” (AAY of 2017.4), is a nonsurgical intervention. Although corneal transplantation surgery for FECD is relatively mature, problems such as graft rejection, skill requirements of EK and the shortage of corneal transplant donors are inevitable. Therefore, a pharmacological approach to treating FECD would be attractive. ROCKs are protein serine/threonine kinases, and the Rho/ROCK pathway is mainly responsible for the modulation of the actin cytoskeleton and the regulation of cell proliferation, migration, and apoptosis [8]. ROCK inhibitors are associated with multiple ocular diseases, such as glaucoma, vitreoretinal diseases and corneal endothelial diseases. In corneal endothelial diseases, ROCK inhibitors could promote the adhesion of corneal endothelial cells (CECs) and restore the pump and barrier function of CECs [28, 29]. In addition, it could modulate both cyclin D and p27, regulate G1/S transition of the cell cycle and promote CECs proliferation [30]. In 2013, Koizumi et al. first used the ROCK inhibitor Y-27632, combined with corneal endothelial denudation, in the treatment of an FECD subject [8]. The subject underwent corneal endothelial denudation followed by topical administration with ROCK inhibitors as eye drops six times daily for one week. Significantly improved vision and decreased corneal thickness were observed by six months and maintained after two years of treatment. ROCK inhibitor was also used as a salvage agent after descemetorhexis without endothelial keratoplasty (DWEK) [31, 32]. FECD subjects, who underwent DWEK and fail to have corneal clearance, were administrated with topical ROCK inhibitor six time daily for two weeks. Corneal clearance and improved visual acuity were observed [31]. In 2018, the combined use of ROCK inhibitors and cultured endothelial cells (CECs) for the effective treatment of bullous keratopathy subjects was reported in the the *New England Journal of Medicine*. [33] The mixture of inhibitors and cultured cells was injected into the anterior chamber of the study subjects. Increased cell density was observed in all treated eyes. As injection of human CECs was already reported to be effective to some extent in FECD subjects, the efficacy of combining CEC use with ROCK inhibitors in FECD warrants further investigation [34]. While the ROCK inhibitor in the treatment of glaucoma has been approved by Food and Drug Administration (FDA), its use in FECD is still at the stage of clinical research [35].

DWEK, research on which was indicated by the keyword “descemetorhexis” (AAY of 2017.4), refers to descemetorhexis without endothelial keratoplasty or descemetorhexis without graft placement. Although EK is relatively mature in the treatment of FECD, complications such as graft rejection and graft detachment could not be avoided. It was reported that only half of EK grafts survived over a 5-year period [36]. In addition, the 5-year graft survival rate was only 77.1% after DSEK [37]. DMEK, though with relatively lower rejection rate than DMEK, the postoperative graft detachment rate was high [38, 39]. A rebubbling technique must be used in some cases to promote graft re-attachment. However, these complications may be evitable by DWEK. Though human endothelial cells were long thought to be unable to replicate, researchers have increasingly proposed in recent years that CECs have mitotic abilities [40, 41]. Therefore, some surgeons have attempted to remove the diseased endothelium without the transplantation of a graft in FECD subjects. Finally, the term “DWEK” was proposed in 2018 [42]. Initially, the postoperative course was not desirable in FECD subjects after DWEK [43]. Prolonged corneal edema was observed in some cases. However, this situation may improve when this procedure is combined with the use of ROCK inhibitor [31]. It was reported that the immediate administration of ROCK inhibitor after DWEK resulted in higher corneal endothelial cell counts and short time to corneal clearance [32]. However, although this operation avoids the problem of graft rejection or detachment, its success rate varies greatly. Clearance rates of the cornea have been reported to range from 63 to 100% [44]. This wide variance may be attributed to the subject’s baseline and the surgeon’s skill [45]. Soh et al. proposed that young age is a critical factor that promotes the migration of CECs [45]. In addition, the removal of an endothelium of a larger size may lead to failure [46]. A descemetorhexis size ≤ 4 mm may be associated with better visual outcomes [47]. On the one hand, DWEK with a descemetorhexis size ≤ 4 mm could lead to improved visual acuity and pachymetry while DWEK with a descemetorhexis size > 4 mm could not. On the other hand, the failure rate of DWEK with a descemetorhexis size ≤ 4 mm was only 4% while the total failure rate was up to 17%.

### Limitations

Although our bibliometric analysis was as comprehensive as possible, some limitations remained. First, as WoSCC was suitable for accurate and comprehensive citation analysis, we extracted data from WoSCC [48]. Therefore, we did not use other search engines, such as Pubmed and Google Scholar, in our analysis. Second, only publications written in English were included in our analysis, which may have induced linguistic bias. Third, some emerging topics without sufficient citations at the time of our analysis may not have been recognized.

## Conclusion

Through this bibliometric analysis, we showed that FECD research has focused mainly on corneal transplantation surgery for the past 20 years. Although transplantation surgery is currently the main treatment option for FECD, several difficulties remain with this approach, such as graft rejection and a shortage of corneal transplant donors. Therefore, a pharmacological approach would be a promising option for FECD treatment. New gene mutations related to FECD are constantly being discovered, and nonsurgical treatments for FECD have emerged. An increasing number of researchers are devoted to exploring gene editing and drug therapy for FECD although approaches remain in the preclinical phase. Among the nonsurgical interventions under study, ROCK inhibitor has been proven to have a positive effect. Perhaps other mature treatment strategies in addition to surgical interventions will become available in the near future.

## Supplementary Information

**Additional file 1:** Data.

**Additional file 2:** **Supplementary Figure 1.** Contributions of different countries to FECD research. FECD: Fuchs endothelial corneal dystrophy.

**Additional file 3:** **Supplementary Figure 2.** Co-authorship analysis of countries. The size of a node is proportional to the number of collaborations.

**Additional file 4:** **Supplementary Figure 3.** Country collaboration map. Two countries connected by a red line indicates that these countries have cooperated with each other. The color intensity of a country is related to the number of collaborations, with a darker color indicating a greater number of collaborations.

**Additional file 5:** **Supplementary Figure 4.** Top 20 institutions with the highest number of publications in the field of FECD research. The x-axis represents the institution’s proportion of the total 1041 publications.

**Additional file 6:** **Supplementary Figure 5.** Co-authorship analysis of authors. The size of a node is proportional to an author’s number of collaborations.

**Additional file 7:** **Supplementary Figure 6.** Top 20 journals with the highest number of publications in the field of FECD research. The x-axis represents a journal’s proportion of the total 1041 publications.

## Data Availability

Dataset analyzed in the current study are available in the supplemental materials which is a spreadsheet named “Data”.
