# Supplementary material for: A 20-year bibliometric analysis of Fuchs endothelial corneal dystrophy: from 2001 to 2020
Source: BMC Ophthalmol. 2022 Jun 8;22:255. doi: 10.1186/s12886-022-02468-x (PMC9175354; doi:10.1186/s12886-022-02468-x)
Supplement: Supplementary file 2 — Additional file 2: Supplementary Figure 1. Contributions of different countries to FECD research. FECD: Fuchs endothelial corneal dystrophy. [file 12886_2022_2468_MOESM2_ESM.docx]

**
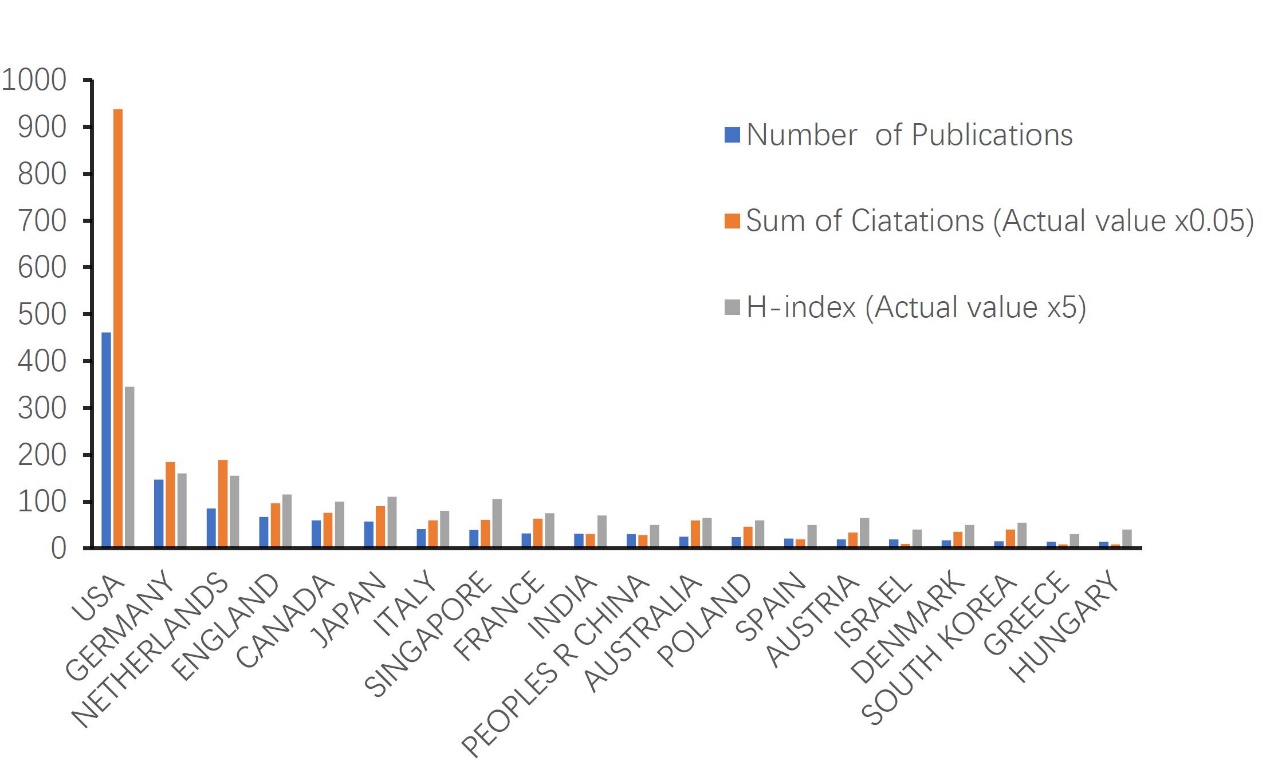
 Supplementary Figure 1. Contributions of different countries to FECD research.** FECD: Fuchs endothelial corneal dystrophy.
