# Supplementary material for: A 20-year bibliometric analysis of Fuchs endothelial corneal dystrophy: from 2001 to 2020
Source: BMC Ophthalmol. 2022 Jun 8;22:255. doi: 10.1186/s12886-022-02468-x (PMC9175354; doi:10.1186/s12886-022-02468-x)
Supplement: Supplementary file 3 — Additional file 3: Supplementary Figure 2. Co-authorship analysis of countries. The size of a node is proportional to the number of collaborations. [file 12886_2022_2468_MOESM3_ESM.docx]

**
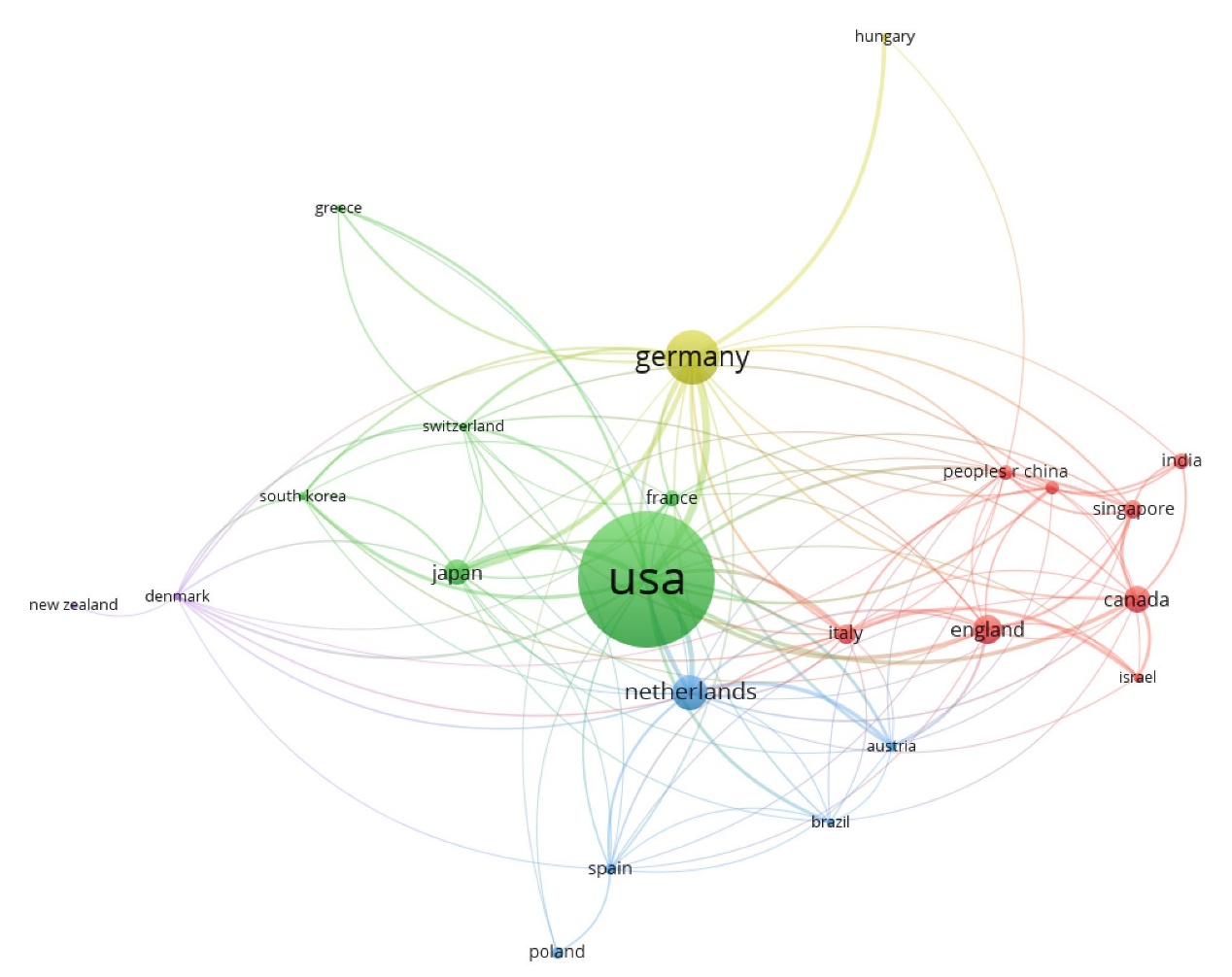
 Supplementary Figure 2. Co-authorship analysis of countries.** The size of a node is proportional to the number of collaborations.
