# Supplementary material for: A 20-year bibliometric analysis of Fuchs endothelial corneal dystrophy: from 2001 to 2020
Source: BMC Ophthalmol. 2022 Jun 8;22:255. doi: 10.1186/s12886-022-02468-x (PMC9175354; doi:10.1186/s12886-022-02468-x)
Supplement: Supplementary file 4 — Additional file 4: Supplementary Figure 3. Country collaboration map. Two countries connected by a red line indicates that these countries have cooperated with each other. The color intensity of a country is related to the number of collaborations, with a darker color indicating a greater number of collaborations. [file 12886_2022_2468_MOESM4_ESM.docx]

**
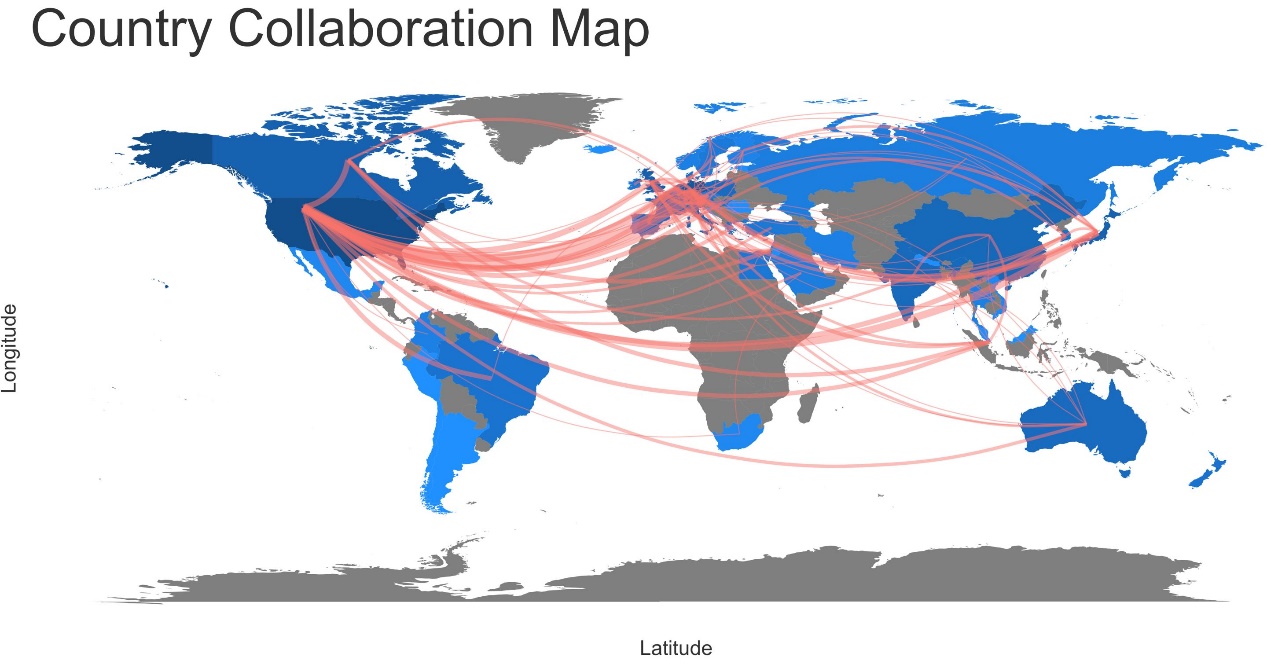
 Supplementary Figure 3. Country collaboration map.** Two countries connected by a red line indicates that these countries have cooperated with each other. The color intensity of a country is related to the number of collaborations, with a darker color indicating a greater number of collaborations.
