# Supplementary material for: A 20-year bibliometric analysis of Fuchs endothelial corneal dystrophy: from 2001 to 2020
Source: BMC Ophthalmol. 2022 Jun 8;22:255. doi: 10.1186/s12886-022-02468-x (PMC9175354; doi:10.1186/s12886-022-02468-x)
Supplement: Supplementary file 5 — Additional file 5: Supplementary Figure 4. Top 20 institutions with the highest number of publications in the field of FECD research. The x-axis represents the institution’s proportion of the total 1041 publications. [file 12886_2022_2468_MOESM5_ESM.docx]

**
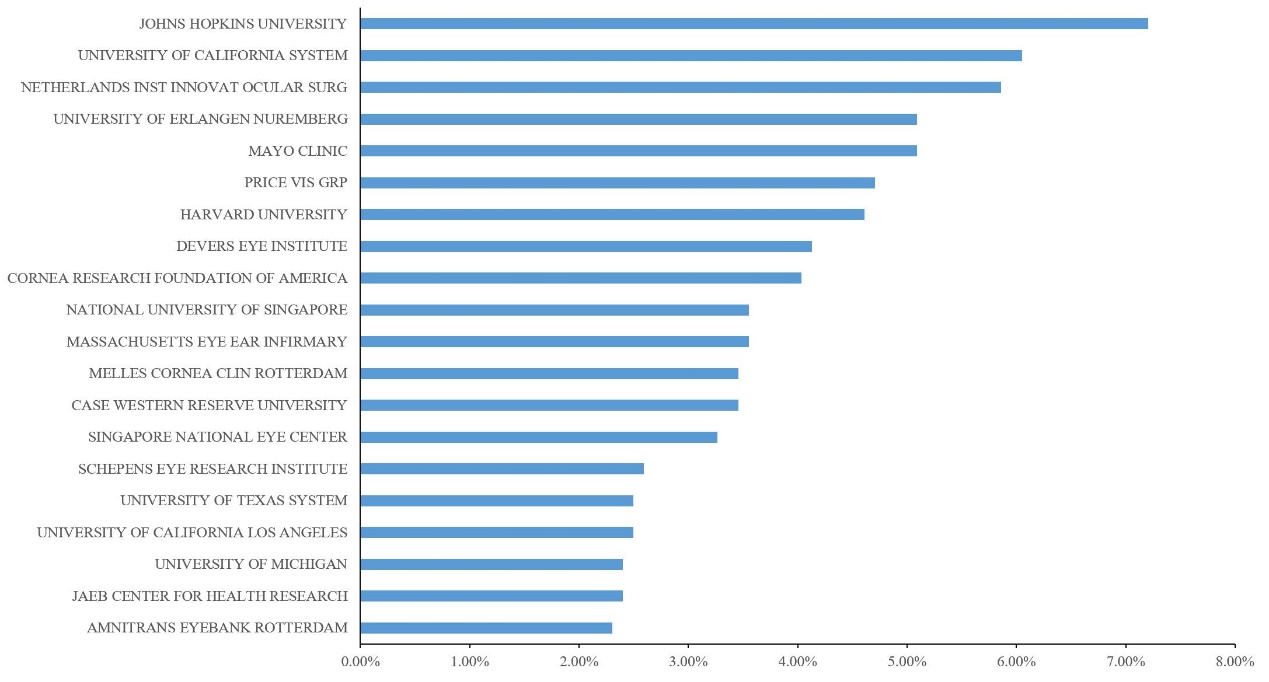
** **Supplementary Figure 4. Top 20 institutions with the highest number of publications in the field of FECD research.** The x-axis represents the institution’s proportion of the total 1041 publications.
