# Supplementary material for: A 20-year bibliometric analysis of Fuchs endothelial corneal dystrophy: from 2001 to 2020
Source: BMC Ophthalmol. 2022 Jun 8;22:255. doi: 10.1186/s12886-022-02468-x (PMC9175354; doi:10.1186/s12886-022-02468-x)
Supplement: Supplementary file 6 — Additional file 6: Supplementary Figure 5. Co-authorship analysis of authors. The size of a node is proportional to an author’s number of collaborations. [file 12886_2022_2468_MOESM6_ESM.docx]

**
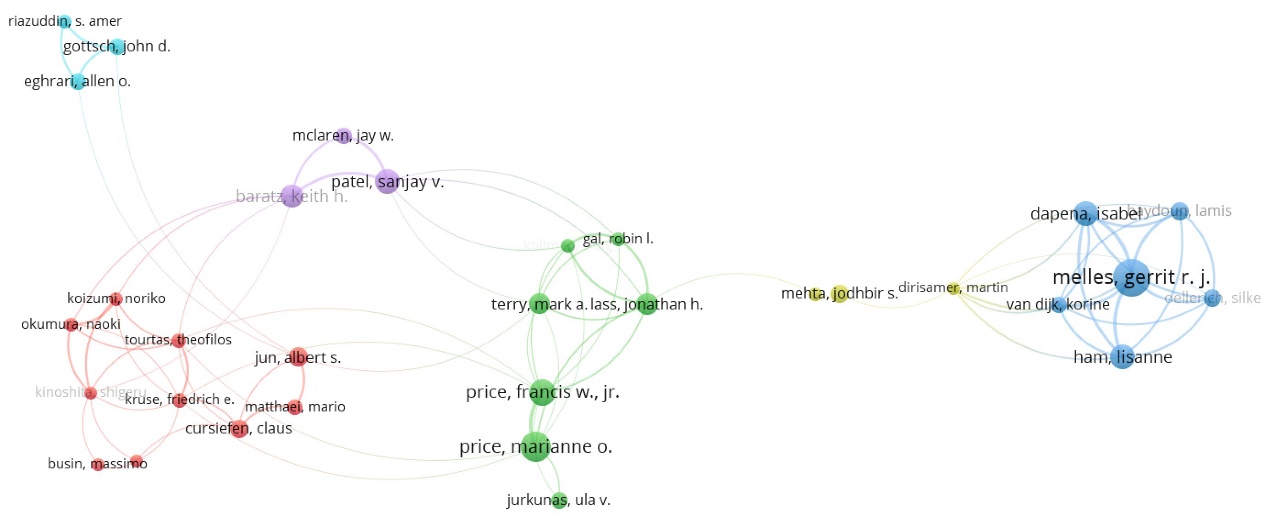
** **Supplementary Figure 5. Co-authorship analysis of authors.** The size of a node is proportional to an author’s number of collaborations.
