# Supplementary material for: A 20-year bibliometric analysis of Fuchs endothelial corneal dystrophy: from 2001 to 2020
Source: BMC Ophthalmol. 2022 Jun 8;22:255. doi: 10.1186/s12886-022-02468-x (PMC9175354; doi:10.1186/s12886-022-02468-x)
Supplement: Supplementary file 7 — Additional file 7: Supplementary Figure 6. Top 20 journals with the highest number of publications in the field of FECD research. The x-axis represents a journal’s proportion of the total 1041 publications. [file 12886_2022_2468_MOESM7_ESM.docx]

**
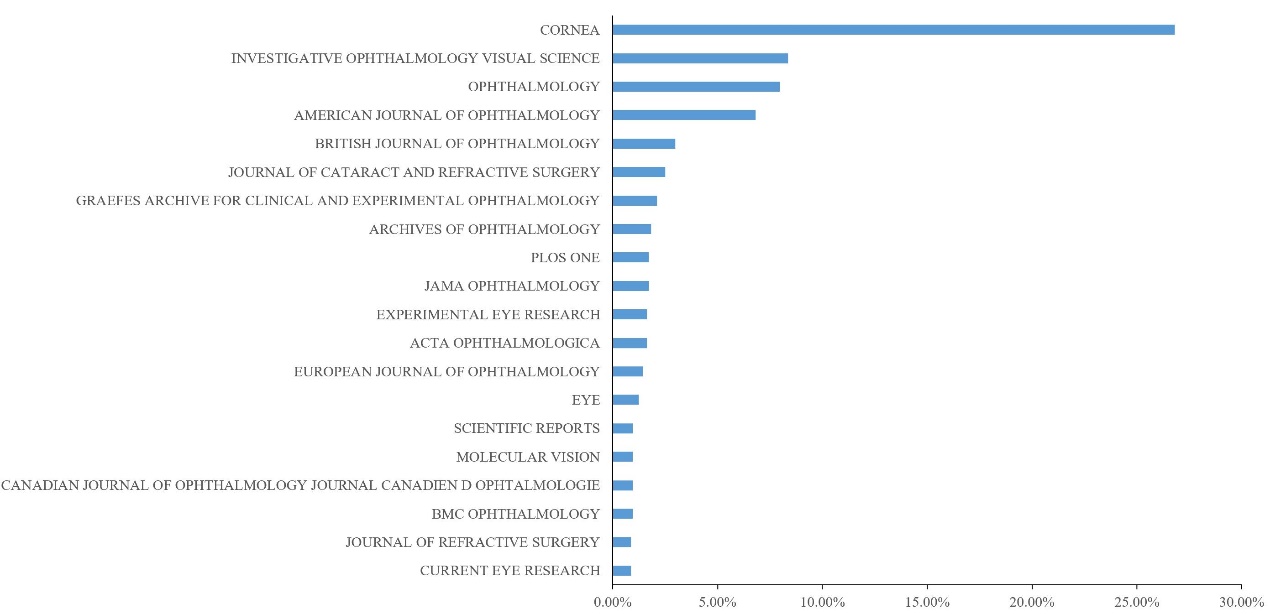
** **Supplementary Figure 6. Top 20 journals with the highest number of publications in the field of FECD research.** The x-axis represents a journal’s proportion of the total 1041 publications.
